# Advancing Insights Into Postoperative Sleep Quality and Influencing Factors

**DOI:** 10.2196/69193

**Published:** 2025-02-03

**Authors:** Yining Zhao, Xin Hu

**Affiliations:** 1 Department of Cardiology Faculty of Medicine The First Hospital of Shanxi Medical University Shanxi China; 2 The First Affiliated Hospital Jiangxi Medical College Nanchang University Nanchang China

**Keywords:** sleep quality, wearable sleep monitoring wristband, intensive care unit, minimally invasive surgery, traditional open surgery

We are writing to provide our comments on the recently published article titled “Quantitative Impact of Traditional Open Surgery and Minimally Invasive Surgery on Patients’ First-Night Sleep Status in the Intensive Care Unit: Prospective Cohort Study” by Shang et al [1]. This paper addresses an important clinical question regarding postoperative sleep quality and provides valuable insights into the advantages of minimally invasive surgery (MIS). We commend the authors for their efforts in using both subjective questionnaires and objective wearable technology to explore this topic. However, we would like to share several thoughts that could help further refine the interpretation of results and consider additional avenues for future studies.

First, this study focuses on postoperative sleep but overlooks baseline sleep conditions. Preoperative sleep disorders or psychological issues, such as anxiety or depression, could predispose patients to postoperative disturbances, while good baseline sleep quality may confer greater resilience [2]. Preoperative assessments using tools like the Pittsburgh Sleep Quality Index or short-term monitoring could help clarify the independent impact of surgical methods. Additionally, pain management significantly affects sleep through both direct and indirect mechanisms. While MIS likely reduces sleep interference due to less pain, the absence of quantitative pain scores and analgesic data limits interpretability. Future research should address preoperative sleep and analgesic strategies to provide a more comprehensive understanding.

Second, wearable devices offer convenience but have limitations in postoperative or intensive care unit settings. They rely on accelerometers and heart rate variability without electroencephalogram support, risking overestimation of sleep duration, especially in patients who are sedated [3]. Environmental factors like intensive care unit noise and caregiving activities can further compromise accuracy, while algorithms optimized for healthy populations may not reflect the sleep patterns of patients who are critically ill. Future studies should improve algorithms, validate accuracy in complex environments, and integrate devices with other monitoring methods to enhance clinical utility.

Third, psychological factors such as preoperative anxiety or depression significantly influence postoperative sleep. Patient perceptions of MIS, such as reduced trauma and faster recovery, may also indirectly improve sleep. However, the study did not quantify these factors, potentially underestimating their role. Additionally, the single-center design and small sample size limit generalizability and subgroup analyses. Future research should adopt multicenter, larger-scale studies and include psychological assessments to enhance applicability and robustness.

In conclusion, Shang et al’s [1] study provides meaningful insights into the impact of surgical approaches on postoperative sleep quality, particularly highlighting the potential benefits of MIS. While the findings are valuable, addressing the aforementioned limitations could enhance the robustness and clinical relevance of future research. We appreciate the authors’ contribution to this important topic and look forward to further advancements in this field.

## Abbreviations

MIS: minimally invasive surgery
